# Inhibition of Angiogenesis Mediated by Extremely Low-Frequency Magnetic Fields (ELF-MFs)

**DOI:** 10.1371/journal.pone.0079309

**Published:** 2013-11-14

**Authors:** Simona Delle Monache, Adriano Angelucci, Patrizia Sanità, Roberto Iorio, Francesca Bennato, Fabrizio Mancini, Giancaterino Gualtieri, Rosella Cardigno Colonna

**Affiliations:** 1 Department of Biotechnological and Applied Clinical Sciences, University of L’Aquila, L’Aquila, Italy; 2 Department of Industrial and Information Engineering and Economy, University of L’Aquila, L’Aquila, Italy; Columbia University, United States of America

## Abstract

The formation of new blood vessels is an essential therapeutic target in many diseases such as cancer, ischemic diseases, and chronic inflammation. In this regard**,** extremely low-frequency (ELF) electromagnetic fields (EMFs) seem able to inhibit vessel growth when used in a specific window of amplitude. To investigate the mechanism of anti-angiogenic action of ELF-EMFs we tested the effect of a sinusoidal magnetic field (MF) of 2 mT intensity and frequency of 50 Hz on endothelial cell models HUVEC and MS-1 measuring cell status and proliferation, motility and tubule formation ability. MS-1 cells when injected in mice determined a rapid tumor-like growth that was significantly reduced in mice inoculated with MF-exposed cells. In particular, histological analysis of tumors derived from mice inoculated with MF-exposed MS-1 cells indicated a reduction of hemangioma size, of blood-filled spaces, and in hemorrhage. In parallel, in vitro proliferation of MS-1 treated with MF was significantly inhibited. We also found that the MF-exposure down-regulated the process of proliferation, migration and formation of tubule-like structures in HUVECs. Using western blotting and immunofluorescence analysis, we collected data about the possible influence of MF on the signalling pathway activated by the vascular endothelial growth factor (VEGF). In particular, MF exposure significantly reduced the expression and activation levels of VEGFR2, suggesting a direct or indirect influence of MF on VEGF receptors placed on cellular membrane. In conclusion MF reduced, in vitro and in vivo, the ability of endothelial cells to form new vessels, most probably affecting VEGF signal transduction pathway that was less responsive to activation. These findings could not only explain the mechanism of anti-angiogenic action exerted by MFs, but also promote the possible development of new therapeutic applications for treatment of those diseases where excessive angiogenesis is involved.

## Introduction

The inhibition of angiogenesis may represent a suitable therapeutic strategy for diseases in which the pathogenesis is sustained by the presence of continuous angiogenic stimuli such as diseases associated with chronic inflammation or aberrant cell proliferation (e.g. tumours). Considering the latest studies reporting that electromagnetic fields (EMFs) and static magnetic fields (SMFs), are able to affect vessel growth and development, both *in vitro* and *in vivo*, the investigation about the modulation of angiogenesis by EMFs and SMFs is becoming increasingly relevant [1], [2], [3]. Static magnetic fields are magnetic fields which do not vary with time (frequency of 0 Hz). They are created by a magnet or by the steady flow of electricity, for example in appliances using direct current (DC). Conversely, EMFs are magnetic field generated by appliances using alternating current (AC) and have frequencies up to 300 Hz. For this reason are also identified as extremely low frequency (ELF) fields.

Although few studies have investigated the anti-angiogenic properties of EMFs, some inhibitory effects on microcirculation and microvasculature have already been demonstrated. For instance, Williams and colleagues reported that pulsed EMFs were able to reduce in vivo the rate of angiogenesis in breast tumours [4]. Another study described that an SMF exerted an anti-angiogenic activity in a chicken-chorioallantoic membrane assay [5]. Several studies have reported that extremely low frequency- electromagnetic fields ELF-EMFs can affect both cell surface receptor expression or activation and downstream signal transduction pathways [6], [7], [8], [9], [10]. These overall findings lead to the hypothesis that the effect of EMFs and SMFs may be propagated and effectively amplified along the whole signal transduction pathway, ultimately modifying cell behaviour. The proliferation of endothelial cells mediated by VEGFR2 requires the ligand-dependent dimerization and phosphorylation, with a subsequent activation of several intracellular pathways, such as Src, PI3K and Raf/MEK/ERK 1/2 [11], [12] and among these, especially ERK plays a pivotal role in correlating the growth factor receptor activation at the cell membrane with cellular signal cascade transduction pathways, which result in transcriptional modification in the nucleus and ultimately in cell proliferation and differentiation [13]. Regarding the hypothesized mechanisms by which EMF could modulate angiogenesis, it is known that EMFs act primarily as stress inducers and mediate cell responses through up regulation of stress proteins, including heat shock proteins (HSPs) [14], [15]. Many reports have demonstrated changes in the expression levels of HSPs as a response to EMF exposure [16] and they have been proposed in risk assessment due to their sensitivity to even minor insult [17]. HSPs expression levels and activity can modify the half-life of many target proteins including VEGFR1 and VEGFR2 [18]. For example it has been described that HSP90 and HSP70 are implicated in both protein stabilization and presentation to degradative pathway [19].

Concerning the electromagnetic stimulations, some studies also found a positive effect of EMFs and SMFs on angiogenesis in vivo and in vitro [2], [20], [21]. For example, we reported that a MF of 1 mT intensity promoted HUVECs proliferation, motility and tubule formation and that VEGFR2 (KDR/Flk-1) was involved in the angiogenic response of HUVECs to MF [21]. This apparent controversy is not surprising because it has been extensively demonstrated that amplitude, frequency and exposure pattern windows of EMFs can influence significantly their biological effects [3], [22], [23]. On this subject, for example, Wei et al. suggested that 60 Hz EMFs at intensities of 90 and 120 mT can increase [3H] thymidine incorporation into DNA of astrocytoma cells in a time dependent manner, while 60 mT have no effect on the DNA synthesis, demonstrating that no or opposite effect could be registered only by modifying the EMF intensity [24], [25]. Considering all these aspects, we aimed to verify whether sinusoidal MF with different field characteristics could modify in specific manner endothelial cell behaviour, positively or negatively, and then we thought of exploring an MF treatment able to inhibit angiogenic potential of endothelial cells in vitro and in vitro. To verify the effect of MF on endothelial cells in vivo we inoculated mouse endothelial transformed cells (MS-1) previously exposed to MF (2 mT, 50 Hz) in C57BL/6 mice. MS-1 cells express VEGFR2 and they are commonly used in the study of signal transduction downstream of angiogenic factors. Moreover, these cells represent the only cell line known that gives rise to benign hemangiomas [26]. Haemangioma are benign tumours of the vasculature frequently encountered in children [27], [28]. Although the pathogenesis of hemangioma formation is poorly understood, angiogenic growth factors and steroid hormone have been postulated to play a pivotal role in hemangioma development [28], [29]. Use of this cell model permits to formulate preliminary hypothesis about a potential therapeutic use of MFs in hemangioma cases.

## Materials and Methods

### Materials

Reagents, when not otherwise specified, were purchased from Sigma Chemicals (St. Louis, MO, USA). Primary antibodies against phospho-ERK1/2, ERK1/2, phospho-VEGFR2 (Flk-1/KDR), VEGFR2 (Flk-1/KDR), and β-actin were obtained from Santa Cruz Biotechnology (Santa Cruz, CA, USA). Primary antibodies against HSP70 and HSP90 were from Enzo Life Science, (Farmingdale, NY, USA). The “In Vitro Angiogenesis Assay” was purchased from CHEMICON International (Temecula, CA, USA). Reagents for enhanced chemiluminescence (ECL) were from Amersham (Amersham Pharmacia Biotech, MI, Italia).

### Cell Culture

Human umbilical vein endothelial cells (HUVECs) were purchased from Lonza (Lonza, Basel, Switzerland) and routinely cultured in endothelial cell growth medium 199 (M199) containing 2 mM glutamine, 100 µg/ml penicillin/streptomycin, heparin (100 µg/ml) and endothelial growth factors (30 µg/ml) and supplemented with 15% fetal bovine serum (FBS). We purchased Mile Sven (MS)-1 cells from ATCC (CRL-2279). The mouse endothelial transformed cell line, MS-1, is a pancreatic islet endothelial cell line transduced with a temperature-sensitive SV40 large T antigen (tsA-58-3) construct. The line retains many properties of endothelial cells, including uptake of acetylated LDL and expression of both factor VIII-related antigen and VEGF receptor. This line was grown in Dulbecco’s modified Eagle’s medium (D-MEM- Sigma-Aldrich, St Louis, MO, USA) supplemented with 5% FBS, 10 nM glutamine and 1% penicillin/streptomicyn. The cells were maintained in a 37°C incubator in a humidified atmosphere containing 5% CO_2_. Cell number was determined after detachment by trypsin by direct cell counting of adherent cells with the use of a haemocytometer, and viability was assessed by exclusion of trypan blue.

### Exposure Conditions

**Figure 1:**
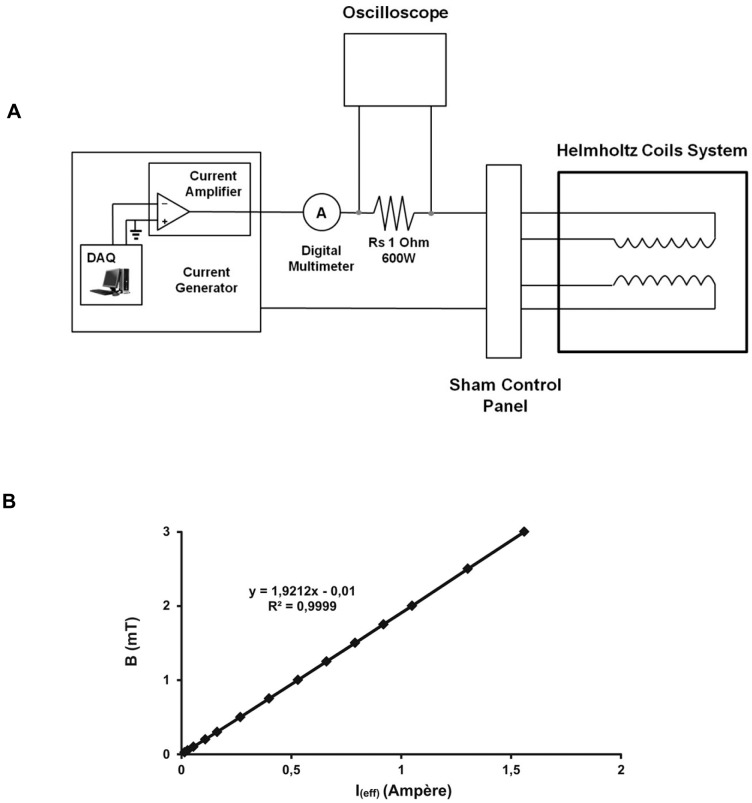
Exposure system. A) Experimental apparatus employed for oscillating magnetic field generation at the centre of the solenoid. B) B versus I(eff) at the centre of the solenoid as experimentally determined. The best fitting straight line of the measured values was obtained using the method of the least squares.

The ELF-MF exposure system consisted of an apparatus (Fig. S1) containing a waveform generator, a current amplifier and a pair of Helmholtz coils (Fig. 1 A). The waveform generator was composed of a PCI DAQ (Digital Acquisition NI PCI 6040E; National Instruments, Austin, TX, USA) placed in a personal computer with the waveform editor software. In our experiments, for the magnetic field generation we employed a pair of Helmholtz coils with mean radius of 13.0±0.5 cm. In each coil the number of turns was 800 with a 2 mm^2^ wire giving a resulting resistance of 2.4 Ω and an inductance of 39±1 mH, as experimentally determined. The mean vertical distance between the coils was 13.5±0.5 cm. According to the different connections, the current could either flow in the same direction or in the opposite direction (sham system), where the magnetic flux density is “theoretically zero” [30]. The uniformity of the magnetic field was better than 1% within a cylindrical region that allowed a simultaneous exposure of a stack of five culture plates (Petri dishes or multiwell plate). This feature was in good agreement with the computation of the field distribution and homogeneity calculated by a Laplace equation simulation program, which took into consideration the finite dimensions of coils. In order to verify the uniformity of the magnetic field in the core of the Helmholtz coils systems, some measurements were performed by using a Gauss meter (Model 912; RFL Industries, Boonton, NJ, USA) connected to a magnetic probe (1 mm^2^). A current generator (BOP 72-6M; Kepco, Flushing, NY, USA) was employed to compensate the effects due to the induced current, and the input current was continuously monitored by means of an oscilloscope (3054 digital oscilloscope, Tektronix, Beaverton, OR, USA) which checked the voltage difference between the 1 Ω (600 W) resistor endings. The rms values of current flowing through the systems were controlled by a Digital Multimeter (Agilent 34401A) (Agilent Technologies, Santa Clara, CA, USA). The systems also have the possibility to have different B values by changing the current in the coils. For this purpose, we found experimentally the B versus I_eff_ (Fig. 1 B), monitoring the current by the shunt resistor connected in series to the coils. The Helmholtz coils system was placed within an incubator at a temperature of 37°C and an atmosphere of 95% air/5% CO2 and 100% relative humidity and it allowed a simultaneous exposure of a maximum of six-culture well-plates. The generated magnetic fields used in this work were sinusoidal waveforms with amplitude of 1 mT (*I*_max_ = 0.530) and 2 mT (*I*_max_ = 1.049) and frequency of 50 Hz. To control the temperature, a thermometric sensor (Fluke 51-II, Fluke, WAQ3) was placed inside the Helmholtz coils system during the experiments measuring a constant temperature of 37.0±0.3°C. In preliminary experiments (sham fields exposure) at 1 mT and 2 mT field intensity we excluded any influences of the experimental devise on environmental parameters such as temperature or gas tension.

For MFs treatments (1 mT and 2 mT) HUVEC and MS-1 were incubated in the presence of sinusoidal MFs for relative treatment times (MF exposed cells) or maintained inside the incubator for the same times (control cells). In particular, HUVECs exposed for 1 h, 6 h and 12 h (exposed cells), unless noted otherwise, after the treatment (1 h, 6 h, 12 h) were maintained unexposed inside the incubator for further 24 h. To synchronize the different treatments and recover the plates at the same time we started with MF exposures sequentially as described:

1*h* = 11*h* of incubation +1*h* of MF treatment;
6*h* = 6*h* of incubation +6*h* of MF treatment;
12*h* = 12*h* of treatment;

For cell growth analysis we prolonged exposure until 24 h so our treatments were as indicated below:

1*h* = 1*h* of MF treatment +23*h* of incubation (without exposure);
6*h* = 6*h* of MF treatment +18*h* of incubation;
12*h* = 12*h* of MF treatment +12*h* of incubation;
24*h* = 24*h* of treatment.

MS-1 cells were exposed or not for overall 72 h and processed immediately for protein extraction or growth analysis assays.

### Growth Analysis Assays

The colorimetric crystal violet technique was used to quantify the effect of MF on endothelial cell proliferation of both cell lines. Briefly, HUVECs and MS-1 were detached from the culture flasks by a trypsin solution (0.05% w/v). Upon cells centrifugation, the pellets were resuspended in specific medium but in the presence of 2% FBS (starved media) and cells cultured for 12 h in order to allow synchronization before treatments. The media were then replaced by the appropriate complete medium as indicated in cell culture section and cells cultured in the presence or in the absence of MF as indicated. These treatments (MF presence or absence) were followed by a further incubation of 24 h at 37°C and 5%CO_2_ in humidified atmosphere.

At the end of incubations, all plates were washed with PBS (3 times), and the cells were fixed for 15 min with 100 µl/well of formaldehyde 3% and sucrose 2% solution. After drying, cells were stained with a solution of 5% crystal violet in 20% methanol for 30 min at room temperature as described. Stain was eluted with a solution of 0.1 M sodium citrate (pH 4.2) and ethanol, and the relative absorbance was stained at 540 nm. Growth of treated samples was determined as a percentage of growth respect to controls.

### Fluorescent Detection of Cellular DNA as a Cell-cycle Phase Indicator

Cells were seeded in 60-mm petri dishes at a density of 3.0×10^5^. After treatments and subsequent incubations of 24 h at 37°C and 5% CO2 in humidified atmosphere, harvested cells were washed and fixed overnight with 70% ethanol. Then, ethanol was removed by centrifugation and the cells resuspended in PBS, stained with the 50 µg/ml propidium iodide (PI) at 4°C for 30 min in the dark. The percent of cells in each phase of the cell cycle (G_1_, S and G_2_/M) was detected by the Tali Image-Based Cytometer (Invitrogen). Phase distributions were calculated from the resultant DNA histogram using FCS Express™ 4 Image Cytometry (Denovo Software) and expressed as a percentage of cells in the respective phases.

### Effects of MF on Tumour Development Histological and Immunofluorescence Analysis

All experimental procedures involving animals and their care were approved by Institutional Review Board and ethic committee of the University of L’Aquila complied with national and international laws and policies (EEC Council Directive 86/609, OJ L 358, 1, Dec. 12, 1987; Italian Legislative Decree 116/92, Gazzetta Ufficiale della Repubblica Italiana n. 40, Feb. 18, 1992; NIH guide for the Care and Use of Laboratory Animals, NIH Publication no. 85-23, 1985). 1×10^6^ MS-1 cells exposed or not to MF and mixed with 0.5 ml of Matrigel added of 50 nM endothelial growth factors (ECGs) were injected subcutaneously into the flanks of the C57BL/6 mice (Charles River, Milan, Italy, 8–12 week old) to induce tumour growth. Before any invasive manipulation, mice were anesthetized with a mixture of ketamine (25 mg/ml)/xylazine (5 mg/ml). During our investigation, the mice were monitored for tumour formation and the time chosen for recovering was based on the tumour size measured using calliper. The observation time was up to 7 days following tumour induction. At termination, tumours were harvested and embedded in optimal cutting temperature compound (O.C.T. compound TISSUE_TEK, Bio-Optica, Milano, Italy) followed by freezing and slicing into 10 µm thick sections at −26°C. Five sections per/group were cut and stained with haematoxylin and eosin and viewed under an optical microscope (Dialux-20-EB, Leitz) and images captured with a digital camera (Nikon) attached to the microscope. Immunofluorescent staining with anti-VEGFR2 polyclonal antibody on cryosections was also conducted. Five different images per slide from three different samples per group with a 200x magnification were randomly analysed. Staining signals were visualized with FITC-conjugated secondary antibodies (Jackson ImmunoResearch Laboratories, West Grove, PA). The sections were counterstained with DAPI and examined using a fluorescent microscope (Zeiss, Axioplan 2).

### HUVEC Migration Assay

Chemotactic motility of HUVECs was assayed using Transwell Assay Plates consisting of six permeable inserts with 6.5-mm diameter polycarbonate filters (8-µm pore size) located in six well receiver plates (Corning Incorporated, Corning, NY, USA) following the protocol provided by the manufacturer. The surface of the filters was coated with gelatine (10 µg/ml) and the media M199 containing 0.5% BSA 1% serum were added to the lower chamber of the filters. Next, cells (1×10^6^ cells/ml) resuspended in this medium deprived of serum were added to the top of each chamber and allowed to migrate through the coated filters for 12 h in the presence or in the absence of MF. Immediately after treatments, cells were assessed for migration rate as follows. Non migrated cells were completely wiped from the top surface of the membrane with a cotton swab while migrating cells adherent to the underside of the filter were stained with Diff-Quick (Baxer Scientific, McGaw Park, IL, USA). Cells were counted at 400x magnification in standard microscopy and the mean number of cells per field was recorded. Five random fields for filters on triplicate filters were used.

### Immunofluorescence Study

To stain phosphorylation of VEGF receptors, we used an anti-phospho KDR/Flk-1 polyclonal antibody (1∶40). Briefly, antibody incubation was carried out at room temperature for 2 h and then cells were washed twice with PBS. After the incubation for 60 min with a secondary antibody mixture (CY3-conjugated rabbit antibody), cells were rinsed, incubated with Hoechst dye 33342 (1 µg/ml) for 5 min, mounted and analysed by confocal microscopy. A minimum of 50 cells/treatment were taken and subjected to image analysis.

### Tube Formation Assay

The tube formation assay in Matrigel was carried out according to the method essentially described by Passaniti et al. [31]. Matrigel was used to coat 96-well plates (50 µl/well) following the manufacturer’s instructions and allowed to solidify at 37°C for 1 h. HUVECs at the concentration of 2×10^4^/well were added to chilled pellets of Matrigel and incubated with medium containing 15% FBS under the three experimental conditions mentioned above (MF exposed and unexposed groups) for up to 12 h. The degree of the angiogenic response was assessed after 16 h from the beginning of the treatment using an inverted phase contrast microscope by evaluating the length of tubes in 10 randomly chosen low-power fields from each well and by counting the number of branching points. Each well was photographed and the relative acquired images quantified using an Image Pro-plus v 4.5 analysis system. On the basis of the two parameters analysed, we obtained the indication to quantify the anti-angiogenic activity, in terms of tubule formation, exerted by MF compared to controls. Mean values and standard deviation (SD) for vessel counts were determined for each analysis.

### Western Blotting Analysis

For western blotting, samples of HUVECs exposed or not to MF for 1 h, 6 h and 12 h were recovered, lysed and separated on 8% SDS polyacrylamide gel under reducing condition and transferred to nitrocellulose membranes.

The immunoblots were blocked by incubation in TBS-T (20 mM Tris-HCl, pH 7.5, 150 mM NaCl, 0.1% Tween 20) containing 5% non-fat milk for 2 h and then incubated with anti-phosphorylated form of VEGFR2 (1∶100) and ERK1/2 (1∶200) as primary antibodies for 2 h at room temperature. The membranes were washed with TBS-T and then incubated with anti-rabbit secondary antibody conjugated to horseradish peroxidase for 1 h. The same blots were stripped by incubation in 62.5 mM Tris-HCl, pH 6.7, 2% SDS, and 100 mM 2-mercaptoethanol for 30 min at 50°C and probed with total forms of VEGFR2 and ERK1/2 (1∶200) to normalize phosphorylation bands. Then, blots were incubated with an anti-mouse IgG horseradish peroxidase and processed for protein determination by enhanced chemiluminescent detection system. Immunoreactive bands were quantified by densitometric analysis.

Samples of MS-1 exposed or not to MF for 72 h were processed for western immunoblot analysis as previously described and detected for the levels of VEGFR2, HSP70 and HSP90.

### Data Analysis

For statistical analysis, all experiments were conducted at least in triplicate and data were presented as means ± SD as indicated. The results were evaluated using ANOVA One Way analysis of variance (ANOVA; Sigmastat 2.03) where otherwise not specified. A *P* value of less than 0.05 was considered statistically significant.

## Results

### Effects of MFs on Endothelial Cell Growth

In a previous work we reported that HUVECs exposed to a sinusoidal MF (1 mT, 50 Hz) showed a marked increase of proliferation rate at 6 h and 12 h of treatment respect to control [21]. In an attempt to verify the existence of a relationship between the observed effects on HUVECs proliferation and duration of applied MFs, we analysed the effect of the sinusoidal MF (1 mT, 50 Hz) on the proliferation rate of HUVECs prolonging cell exposure until 24 h. The treatment was followed by further incubation of 24 h before the growth determination.

**Figure 2:**
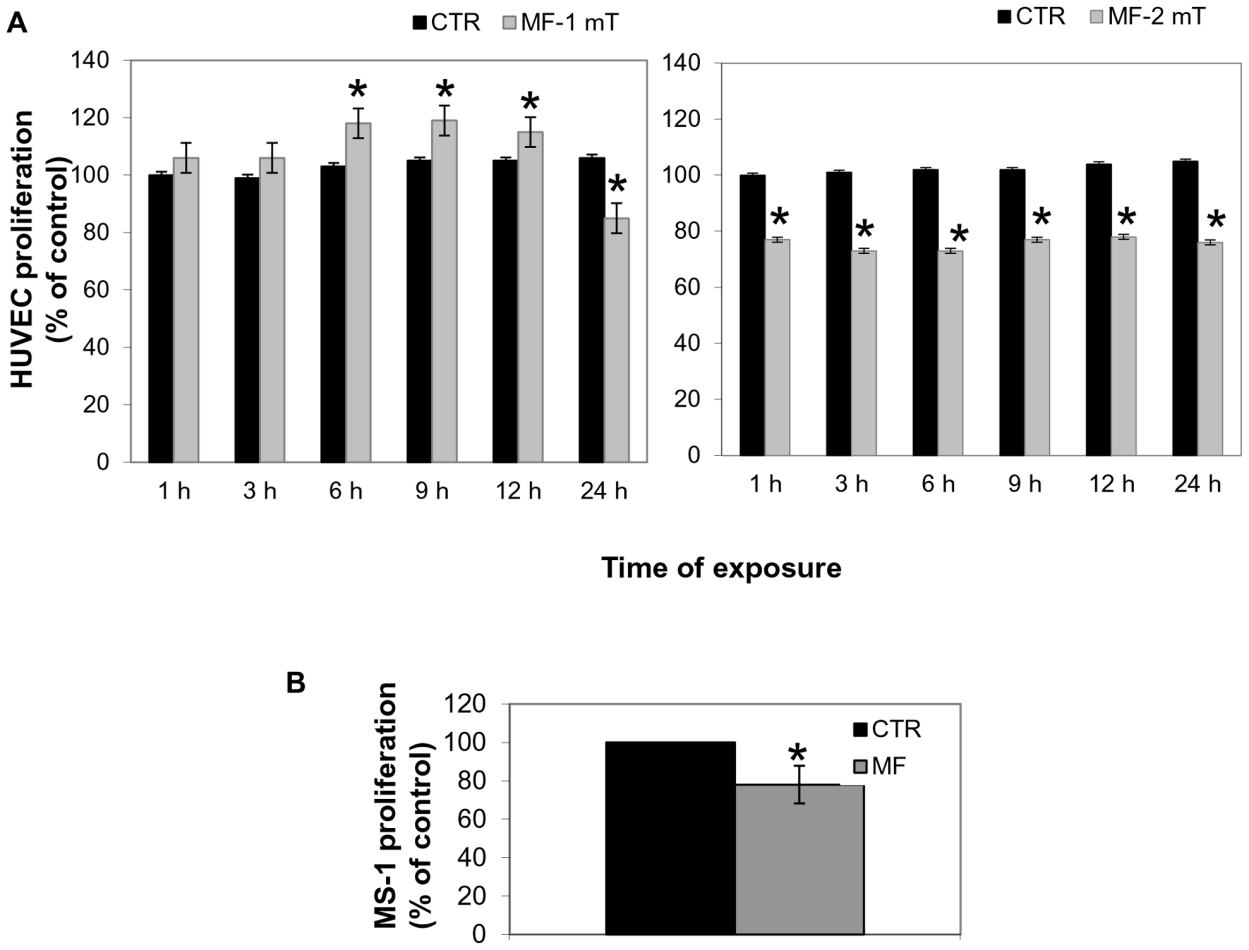
Effect of MF on the proliferation of HUVECs. A) Histograms show the proliferative rate of HUVECs exposed to MFs. Each rate value was calculated respect to CTR at 1 h. The left histogram shows effects of a MF of 1 mT intensity from1 h to 24 h of treatment. The right histogram shows the effects of a MF of 2 mT intensity from 1 h to 24 h of treatment. B) Histogram shows the growth rate of MS-1 cells exposed or not to MF (2 mT, 50 Hz) for 72 h. Statistical differences were indicated for P<0.05.

Previous results were confirmed and the prolonged exposure until 24 h determined a clear arrest of HUVECs growth (Fig. 2 left panel). On the basis of this unexpected result suggesting the possibility for MF to negatively modulate cell growth, we found interesting to explore the effect of MF at increased field intensity. Thus we exposed HUVECs to a sinusoidal 2 mT intensity and frequency of 50 Hz for 1 h up to 24 h determining their effect on cell proliferation at the same endpoint, 48 h. Figure 2a (right panel) shows that MF (2 mT, 50 Hz) resulted in a significant decrease in proliferative rate of HUVECs compared to controls at all times points P<0.05.

Next, we tested the effects of MF (2 mT, 50 Hz) using a transformed murine endothelial cells (MS-1) line. We reproduced a similar experiment for MS-1 and we demonstrated that, after a chronic exposure of 72 h, MF (2 mT, 50 Hz) determined a marked decrease of cell proliferation level when compared with control cells (Fig. 2 B).

### Effects of MF on the Cell Cycle Distribution in HUVECs and MS-1 Cells

To test whether MFs determined cell cycle perturbations, the percentage of cells in each phase of the cell cycle was measured by the Tali Image-Based cytometer. The percentage of HUVECs in the G1, S, and G2/M phases for the control cells and the cells treated with MF for 1 h, 6 h, 12 h and 24 h was reported in the table 1. Analyses were conducted after further 24 h of incubation after the MF treatment. MF caused an increase in the percentage of G2/M phase cells. In fact, the percentage of cell in G2/M phase increased from 29% of the control to 51%, 59%, and 61% in HUVECs treated for 1 h, 6 h and 12 h respectively. In parallel a significant decrease in the percentage of cells in G1 phase was observed. Conversely, the percentage of cells in G2/M phase in HUVECs for 24 h increased at 41%. These results suggested that the arrest of cells in G2/M phase induced by MF was probably due to a transient effect, which was partially recovered during the time. A similar cell cycle analysis was conducted in MS-1 cells (Table 1). In this case, MS-1 cells exposed or not to MF for 72 h were immediately processed to determine the percentage of cells in each phase of cell cycle. Results showed a little increase of cells in G2/M phase shifting from 25% of the control to 34% of MF treated cells.

**Table 1:** MFs effects on cell cycle progression.

	HUVECs					MS-1 cells	
Cell cyclephase	CTR	1 h	6 h	12 h	24 h	CTR	72 h
**G1**	42%	21%	22%	26%	31%	39%	22%
**S**	30%	18%	19%	23%	28%	34%	34%
**G2/M**	29%	51%	59%	61%	41%	25%	34%

Quantitative analysis of cell cycle in HUVECs and MS-1 cells treated with MF for different times.

### MF Inhibited HUVECs Cell Migration

The migration of endothelial cells is a prerequisite for angiogenesis. To investigate the effect of MF (2 mT, 50 Hz) treatment on HUVECs chemotactic ability, human endothelial cells suspended in serum free medium were seeded in chemotactic Transwell plates, subjected to MF exposure for 1 h, 6 h and 12 h and the number of cells that migrated through the polycarbonate filters in the overall time of 12 h was determined as described in materials and methods. Cells exposed to MF for 1 h and 6 h were maintained in incubation until 12 h.

We found that 2 mT MF efficiently reduced the number of migrated cells in the lower face of the filter respect to unexposed control cells. In particular, the MF treatment was able to produce a significant inhibition of HUVECs motility already after 6 h of exposure reaching a statistical value of inhibition of about 21% (P = 0.005). Prolonged exposure of cell until 12 h maintained a similar inhibitory trend (Table 2).

**Table 2:** MFs effects on HUVEC migration.

	MF	CTR	*P*
	Mean ± S.E. n = 7	Mean± S.E. n = 7	*MF vs CTR*
**1 h**	70.25±14.86	67.50±25.9	0. 886
**6 h**	97.13±4.41	122.25±4.20	0.005
**12 h**	147.25±3.57	185.50±12.38	0.002

r.a. = relative absorbance of HUVEC number; n = number of experiments.

Data were analyzed and *P* values calculated using ANOVA One Way analysis of variance.

Mean number ± standard error (S.E.) of HUVECs migrated in Transwell plates in the presence or in the absence of MF (2 mT, 50 Hz) for 1 h, 6 h and 12 h.

### MF Attenuated Capillary Tube Formation of HUVECs

**Figure 3:**
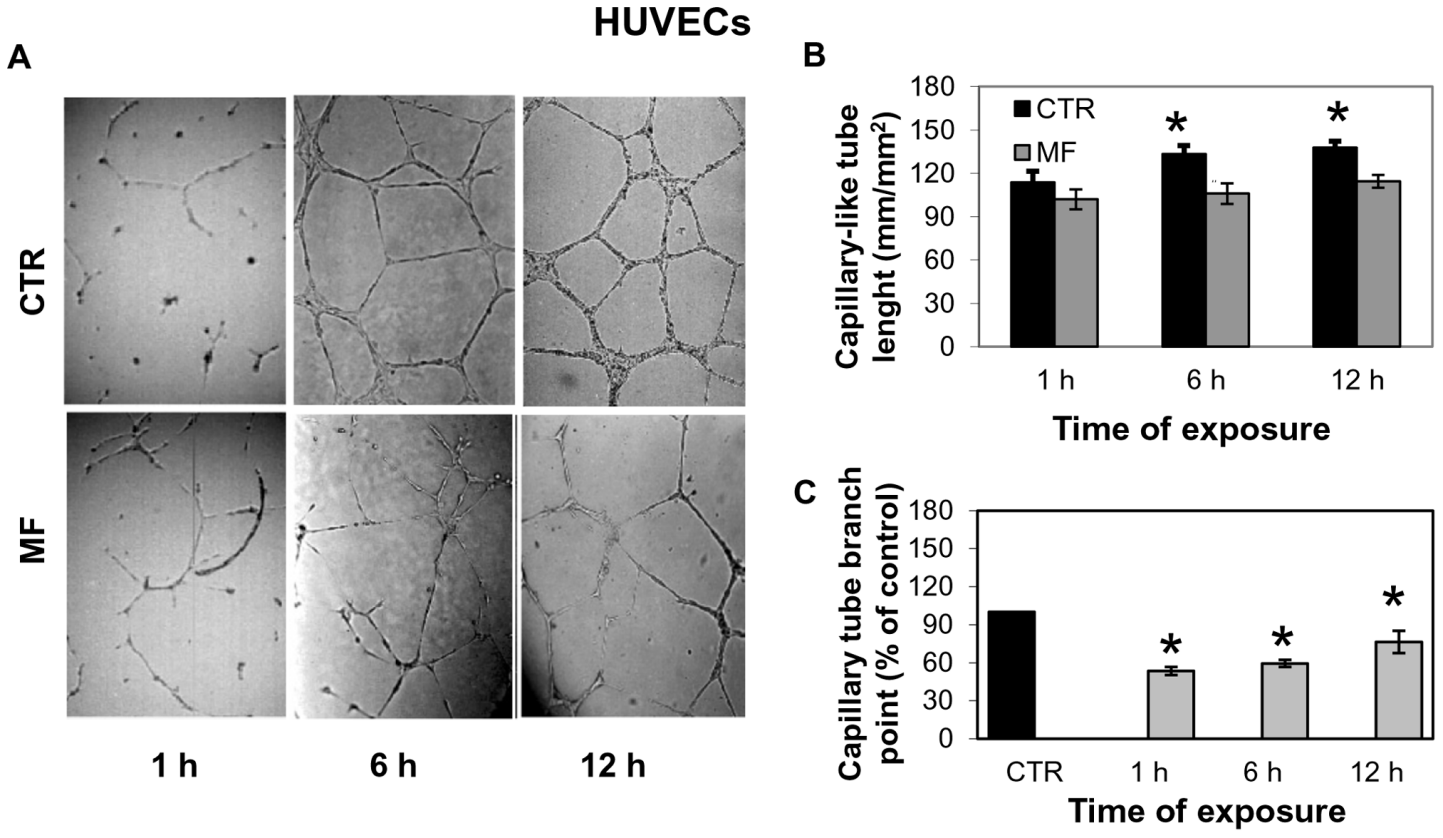
Effect of MF on tubule formation of HUVEC cells. A) Effect of MF (2 mT, 50 Hz) on angiogenesis in vitro. HUVECs were plated on Matrigel-coated culture plates, exposed for 1 h, 6 h and 12 h to MF (D, E, F) and photographed 16 h after plating. Unexposed control cells (CTR) were represented by HUVECs incubated in the absence of any stimulation for 1 h, 6 h and 12 h (A, B, C). B) Quantitative analysis for the tube length n = 5/treatment; C) Quantitative analysis of images for the branch point. n = 5/treatment CTR = unexposed control cells; MF = MF exposed cells. Data were expressed as a percentage of MF respect to unexposed control cells. Statistical differences were indicated for P<0.05.

To verify if MF (2 mT, 50 Hz) affected tubule-like structures formation in vitro, HUVECs were seeded on Matrigel coated plates, exposed to MF for 1 h, 6 h and 12 h and photographed 16 h after plating. We noticed in coated-plates exposed to MF that the capillary tube-like network formation was evidently inhibited (Fig. 3A) respect to controls. The quantitative analysis of images reveals that MF significantly reduced the average tubule length as compared to the control cells already after 6 h from the beginning of exposure (Fig. 3B) and the 6 h exposure determined the major level of inhibition (24%) evaluated by tubules length, respect to control cells (*P* = 0.05). MF treatment also induced an approximate 35% decrease in the number of branch points with respect to the control at all time points (Fig. 3C)”.

### MF Affects MS-1 in vitro and in vivo

**Figure 4:**
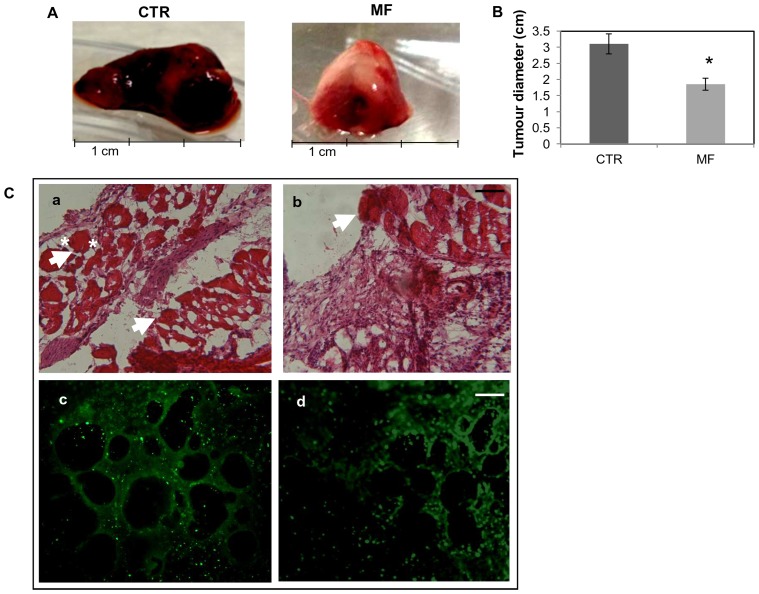
Inhibition of tumour size in mice inoculated with MS-1. A) MF (2 mT, 50 Hz) exposed cells (right) in comparison with unexposed cells (left). B) The size of the tumour was reduced in mice inoculated with MF treated MS-1 cells compared to control mice (inoculated with control cells). N = 5 mice per group, * P<0.05. C) Histology of tissue samples from a mouse inoculated with control cells showed a vascular tumour with large blood-filled lumens (a). The density and size of vessels is reduced in samples from mouse bearing MF-treated cells (b). White arrows indicate some blood filled structures. White asterisks indicate a blood filled diameter. Bar = 0.1 mm.VEGFR2 immunofluorescence micrographs of tissue sections from mice inoculated with control (c) and MF treated cells (d) Bar = 0.2 mm.

To test the effects of MF in a murine model of angiogenic growth, transformed murine endothelial cells MS-1 were utilized. In our experimental model, all mice inoculated with treated or not MS-1 cells (1×10^6^cells) developed an intense endothelial proliferation visible as tumour mass at the site of injection starting from 3–5 days after cell inoculation. At the end point, 7 days after cells injection, in the group of mice inoculated with MF-treated cells (right panel Fig. 4A), tumours were smaller in diameter (average 20 mm) compared to control cells (average 32 mm) (left panel Fig. 4A, D). Histological analysis of the vascular tumours in the control group revealed large blood-filled cavities (Fig. 4B, a). Although blood-filled cavities were also observed in tumour sections of mice inoculated with MF-treated cells, these cavities were strongly reduced in number and partially in size (Fig. 4B, b; Table 3).

**Table 3:** Analysis of blood-filled cavities.

	Injected CTR cells	Injected MF-treated cells	*P* MF vs control
**Blood-filled cavities number**	13.7±1.15	4.3±2.52	0.005
**Blood-filled cavities size (mm)**	0.14±0.024	0.07±0.004	0.01

Mean number and mean size ± standard error (SE) of blood filled-cavities of tumour mice injected with control cells vs MF-treated cells. MF treatment (2 mT, 50 Hz) has been conducted for 72 h.

We investigated the presence of endothelial marker fetal liver kinase (Flk-1) also known as vascular endothelial growth factor receptor-2 (VEGFR2) in tissue sections of mice bearing MS-1-induced vascular tumours. VEGFR2 staining (green fluorescence) was strongly detected in sections of mice bearing not exposed MS-1 (c) while staining for this marker was reduced in sections of mice containing MF- treated MS-1 cells (d) (Fig. 4B c, d).

### MF Effects on VEGFR2 Activation and Downstream Signalling Pathways

**Figure 5:**
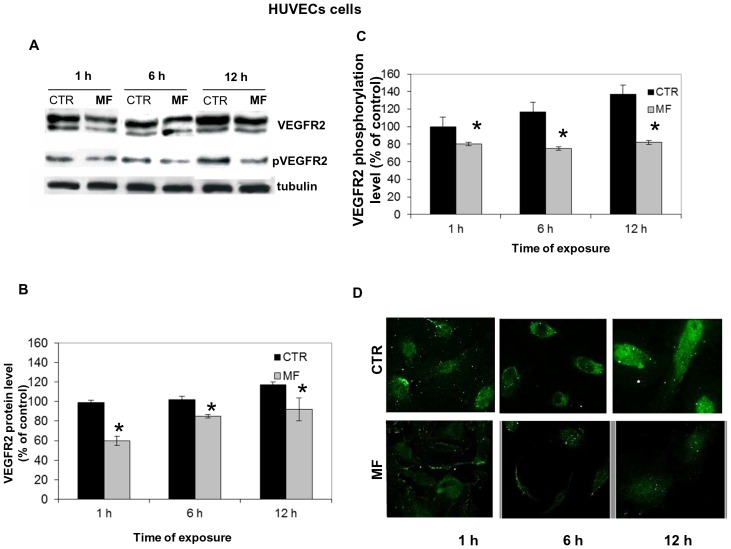
Effect of MF (2 MT, 50 Hz) on VEGFR expression and activation. Protein of equal quantity was separated by 8% SDS-page and electroblotted on to a PVDF membrane. Representative blots show effects exerted by MF (2 mT, 50 Hz) on VEGFR2 total expression and phosphorylation after 1 h, 6 h and 12 h of exposure A). Histograms show values obtained by means ± SD of relative protein bands calculated in 3 experiments after incubation with anti-VEGFR2 antibody B) and with anti-pVEGFR2 antibody C). All protein bands were verified using an anti-tubulin antibody. Asterisks indicate statistical differences * P<0.05 D) Immunofluorescence of HUVECs exposed (bottom panels) or not (upper panels to MF for 1 h, 6 h and 12 h. Phosphor-VEGFR2 receptors were stained with a secondary antibody FITC-conjugated. Images shows that after MF exposure, the density of the phosphorylated receptors resulted decreased at each time point investigated.

Given the central role played by VEGF signalling in angiogenesis [11], [32], [33], we tried to clarify the involvement of the VEGFR2 signalling in MF-induced anti-angiogenic effect. To this purpose, we first investigated the protein expression and the phosphorylation level of VEGFR2 receptor since it results as the central target of VEGF signalling cascade [12], [34]. By western blotting analysis, we showed that MF exposure (1 h, 6 h, 12 h) significantly inhibited the overall VEGFR2 protein levels at any time points (Fig. 5A and 5B), as well as the VEGFR2phosphorylation level of HUVECs (Fig. 5A and 5C). The phosphorylation status of these receptors was also evaluated by immunofluorescence (Fig. 5 D).

**Figure 6:**
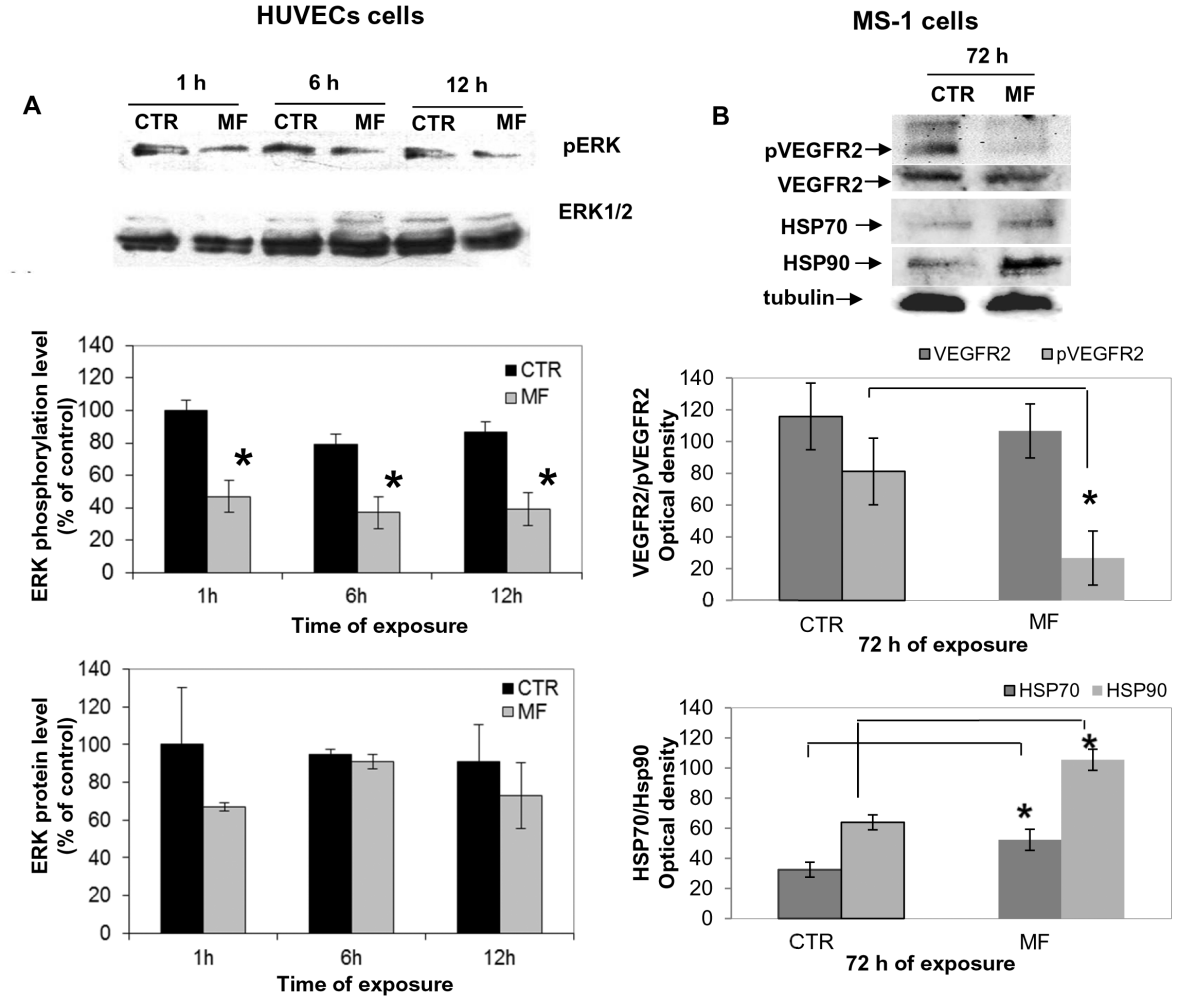
Effect of MF (2 mT, 50 Hz) on ERK activation and phosphorylation levels of HUVECs. A) Representative blots of ERK expression and phosphorylation after 1 h, 6 h and 12 h of exposure to MF of HUVECs. Histograms show values obtained by means ± SD of relative protein bands calculated in at least 3 experiments after incubation with anti-pERK antibody, anti-ERK1,2 antibody. MF = MF exposed cells; CTR = control unexposed cells. Analysis of protein expression levels of MS-1 cells (B) Representative blots of VEGFR2 phosphorylation and expression showed a marked decrease of VEGFR2 active form combined with a more slight reduction of VEGFR2 total form. HSP70 and HSP90 expression after 72 h of exposure to MF in MS-1 cells seemed markedly reduced. Histograms show values obtained by means ± SD of relative protein bands calculated in 3 experiments after incubation with anti-VEGFR2 and anti-pVEGFR2 antibodies and HSP70 and HSP90 antibodies. All protein bands were verified using an anti-tubulin antibody.

As a second step, we detected the levels of expression and phosphorylation of ERK1/2, because ERK signalling cascade results mainly associated with mitogenicity and proliferation [35], [36], and also a reciprocal regulation between VEGF and ERK phosphorylation status has been suggested [37]. HUVECs exposure to MF inhibited the activation of ERK1/2 with a sustained reduction of phosphorylation status until 12 hours of treatment (Fig. 6A). However, we did not observe any significant suppression of the total expression levels of ERK1/2 in MF exposed cells at any time investigated (Fig. 6A, histograms).

Similarly to HUVECs, also MF-exposed MS-1 cells demonstrated the inhibition of VEGFR2 activation (Fig. 6B) and a slight decrease of VEGFR expression. In order to explore the possibility of a stress response related-effect of MF on endothelial cells, Hsp70 and Hsp90 levels were detected on these cells exposed or not to MF for 72 h. As regards VEGFR2 status, we observed a significant increase of expression of these stress proteins which seemed positively modulated by MF (Fig. 6B, histograms).

## Discussion

Recently, the study of EMFs effects on angiogenesis models has much caught the attention of the scientific community for its potential therapeutic value in many diseases. In particular, regarding EMFs effects on living organisms**,** they may behave as inhibitors of the tumour growth, either alone [3], [38], [39] or in combination with anti-angiogenic drugs [4], [40], [41]. In these studies, the observed limitation of tumour development can be attributable to a cytostatic effect directly exerted by EMFs on cancer cells or to an indirect effect on the proliferation of endothelial cells involved in the process of new capillary formation needed to develop and support tumour growth.

In this paper we provide new evidence about the capacity of sinusoidal MFs to affect endothelial cells proliferative status. In fact, we observed a significant decrease in the proliferative rate of HUVECs exposed to a 2 mT MF compared to controls at all times points investigated. This result was unexpected and in contrast to the positive effect we previously observed on HUVECs growth exposed for up to 12 h to a sinusoidal MF of 1 mT intensity.

These contradictory results could be explained by the hypothesis of a “biological window” of MF effects and, in particular, with the “amplitude window” hypothesis [42]. According to this theory, the suggested existence of “specific permitted” levels which bio systems could attain under the action of MF is related to the metabolic status of the system. Moreover, the response of biological systems which are in different metabolic conditions can also result dissimilar. Given the heterogeneity in a cell culture, in which a balance between proliferation and apoptosis exists, if MF affects only a small portion of cells, probably those in a particular metabolic status or in a specific step of their cell cycle, the duration of exposure to MF could considerably change the recorded effects. The evidence that the MF treatment (2 mT) induced cell cycle arrest but not apoptosis is compatible with a progressive adaptation of cell culture to stress. In parallel, the different effects observed increasing MF intensity or increasing the duration of exposure to an MF of 1 mT intensity until 24 h could be dependent on the stimulation of a larger population of cells.

Next, investigating the inhibitory effect of the sinusoidal MF (2 mT, 50 Hz) on HUVECs migration and tubule formation, as essential and critical step in angiogenesis, we demonstrated the ability of 2 mT MF to produce a significant decrease of angiogenesis in vitro. We also confirmed the anti-angiogenic potential of MF (2 mT, 50 Hz) in vivo using a matrigel plug assay with transformed murine endothelial cells exposed to MF (2 mT, 50 Hz). Regarding the possible mechanism underlying the effects of MF (2 mT) on angiogenesis, we found a negative effect of MF on some initiator and/or intermediate elements of the VEGF-dependent signalling pathway which could mediate the effects of various extracellular stimuli to regulate proliferation and differentiation. The possibility that VEGFR2 could play a central role in the biological response of endothelial cells to MF, has already been hypothesized and further strengthened by present results showing that both VEGFR2 expression and activation were prevented in HUVEC exposed to MF (2 mT, 50 Hz).

Angiogenesis is regulated by the control of several factors that in turn can induce (VEGF, FGF, TGF-α) or inhibit (PDGF, endostatin, angiostatin) this process and by numerous intermediate elements of the VEGF signalling pathway such as VEGFR2 and ERK [43], [44]. It has been speculated that cell membrane receptors of cell could be the primary interaction site of EMFs signals and it has been proposed that an interference of an EMF with membrane-mediated signal detection, transduction and biochemical amplification may determine many biological field effects [45], [46]. However, it is not possible to exclude that this inhibition was merely related to the MF-mediated reduction of VEGFR2 proteins available to phosphorylation, which in turn could be caused by specific or not specific influence of MF on this receptors.

We confirmed the hypothesis that MF could affect VEGFR2 in HUVECs by impairing intermediate elements downstream the VEGF signalling pathway such as ERK1/2, which is involved in the regulation of many cellular functions including VEGFR2 gene expression [46]. Besides, ERK is thought to be the most important mediator of VEGF signalling pathway associated with mitogenity, proliferation and migration [35], [36] and EMFs can influence numerous extra-cellular signal regulated kinases including the ERK family [9]. From our results, the exposure of HUVECs to MF inhibited the activation of ERK1/2 with a sustained reduction of phosphorylation status at all times points investigated, while no significant suppression of the total expression levels of ERK1/2 was observed. Thus, the inhibition of active form of ERK1/2 at 1 h of exposure suggested that MF could directly interrupt the signalling pathway mediated by VEGFR2 at level or before ERK activation. In any case, blockade of ERK1/2 activation could be responsible for the decrease of inhibition effects on the process involved in early steps of the angiogenic process, including proliferation, migration and tubule formation [37].

Present results could be interpreted postulating that the “antiangiogenic” action of 2 mT magnetic field on HUVEC, the inhibition of VEGF activated signalling pathway and the decrease in activation of pro-survival ERK pathway, were a stress-related response to radiation [47].

In this way the possible adverse effect of 2 mT MF, not related to heating, on endothelial cells could result in a stress response. To address this issue cell cycle analysis in HUVEC and MS-1 cells was conducted in these experiments we demonstrated that MF determined a cell-cycle delay, probably, by a “transient” arrest of MF-treated cells in G2/M phase. These results suggest that the inhibition of proliferation and the decrease of migration could be also the result of a perturbation in the progression through cell cycle. Our findings are in agreement with other studies demonstrating that numerous stress factors (for example heat) are responsible for an increase of heat shock proteins (i.e. hsp70) correlated to an arrest in G2/M phase. To investigate if cell cycle perturbation by MF could be coupled to a stress response we focused preliminary on the major stress protein Hsp70 and Hsp 90. A panoply of stimuli, including anticancer agents, induces the synthesis of stress-inducible HSPs, which enhance the ability of the cell to survive those otherwise lethal conditions [48] acting as anti-apoptotic agents. Several studies have found that magnetic fields can elevate HSP70 expression or gene activity in cell culture also within 30 min from onset of EMF exposure [49], [50]. Moreover, it is well established that Electromagnetic fields (EMF), in both ELF (extremely low frequency) and radio frequency (RF) ranges, activate the cellular stress response, a protective mechanism that induces the expression of stress response genes, e.g., HSP70, and increased levels of stress proteins, e.g., Hsp70 [48], [51], [52]. Furthermore, HSP70 has also been shown to act at the pre-mitochondrial stage by inhibiting stress-activated kinases and, in apoptosis induced by hyperosmolarity, Hsp70 has been found to modulate JNK and ERK phosphorylation [53], [54].

In previous experiments an influence of 50 Hz MF was described, in the similar range of intensity used in this study, onto Ca^2+^ and K^+^ ion channels [51]. This may suggest that Ca^2+^ and K^+^ channels might be the primary target for the MF action, and this may lead to the activation of Na^+^/K^+^ ATPase in order to restore ion homeostasis, with consequent ATP consumption. Therefore, we postulate that the inhibition of VEGFR2 phosphorylation observed in our results could be due to an uncoupling of Hsp70/Hsp90 complex which requires ATP for its dynamically stabilized conformation. Consequently, the increase of Hsp70 and Hsp90 determined by dissociation of Hsp70/Hsp90 complex could, for example, stimulate the recruitment of Hsp70 to VEGFR2 and increase degradation by such a pathway [19].

In conclusion, our findings indicate that a sinusoidal MF of 2 mT intensity and 50 Hz frequency is able to interfere with the process of angiogenesis inhibiting key molecular factors involved in the activation of VEGFR2. One hypothesis includes the participation of heat shock proteins Hsp70 and Hsp90, which have previously been demonstrated to have a fundamental role in regulating the stability and turnover of VEGFR2 and subsequent VEGF-A regulated responses. Perturbation of HSP70/HSP90 machinery may be attributed to MF effects on Ca^2+^ and K^+^ channels. In fact, inhibition of vessel growth observed after injection of MF-exposed cells in mice suggests that MF could determine a modification of the protein and/or gene expression pattern on exposed cells. MF-induced modification was sufficiently stable to manifest its anti-proliferative effect in cell in inoculated in mice. This aspect indicates that a direct exposure in vivo could be even more evident in inhibiting angiogenesis.

Present data could suggest a promising use of EMFs as potential anti-vascular agents and encourage new therapeutic approaches for the treatment of mammalian tumour, even though further studies on the molecular mechanisms underlying the action of the EMF as antiangiogenic agent are needed.

## Supporting Information

Figure S1 **Photo of Exposure System employed.**
(TIF)
