# Supplementary figures and images for: Inhibition of Angiogenesis Mediated by Extremely Low-Frequency Magnetic Fields (ELF-MFs)
Source: PLoS One. 2013 Nov 14;8(11):e79309. doi: 10.1371/journal.pone.0079309 (PMC3828379; doi:10.1371/journal.pone.0079309)

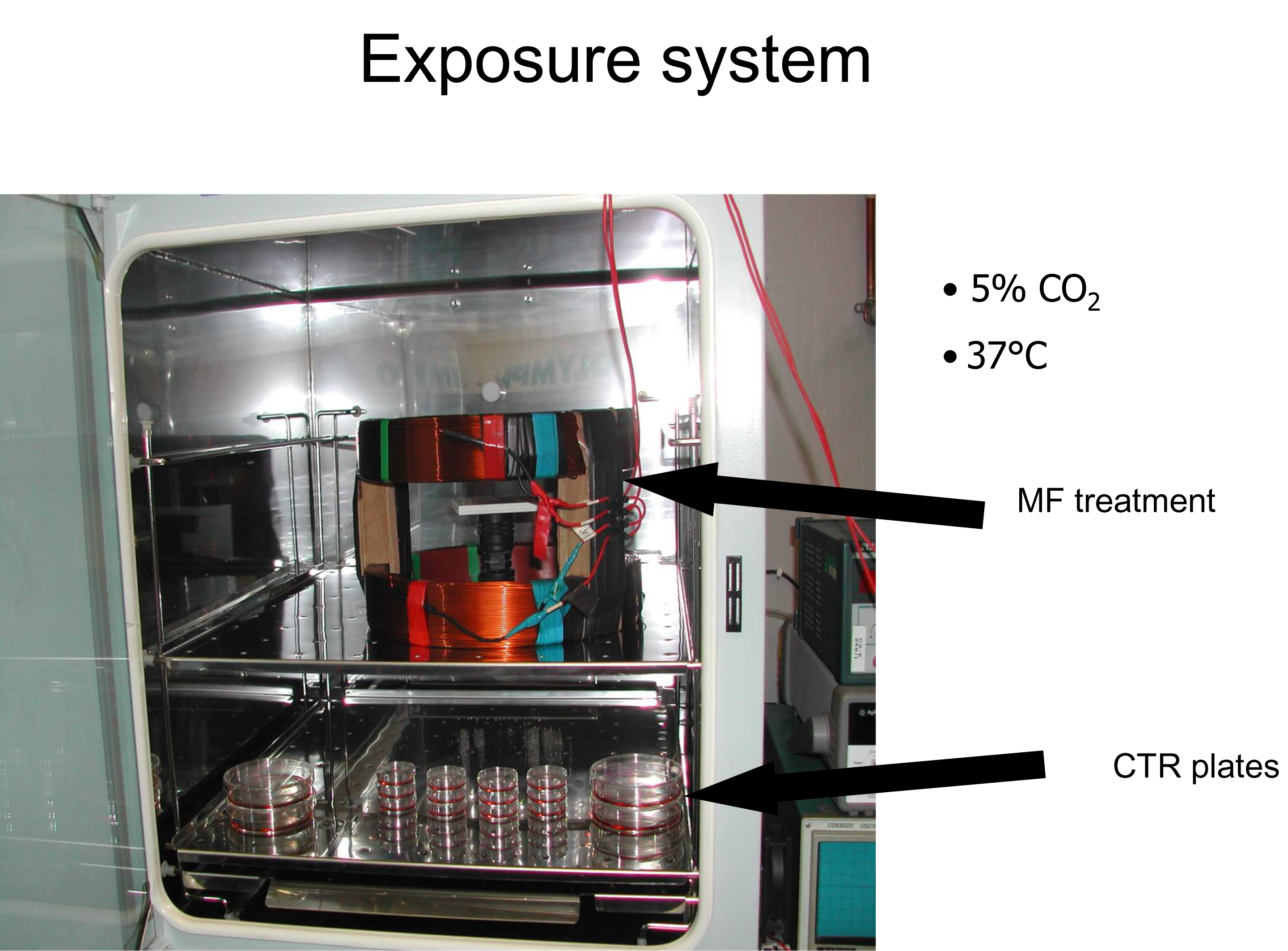

Supplement: Figure S1 — Photo of Exposure System employed. (TIF) [file pone.0079309.s001.tif]
